# The effect of an adapted Mindfulness-Based Stress Reduction program on mental health, maternal bonding and birth outcomes in psychosocially vulnerable pregnant women: a study protocol for a randomized controlled trial in a Danish hospital-based outpatient setting

**DOI:** 10.1186/s12906-023-04194-3

**Published:** 2023-10-14

**Authors:** S Skovbjerg, A Sumbundu, M Kolls, A Kjærbye-Thygesen, LO Fjorback

**Affiliations:** 1 Danish Center for Mindfulness, Department of Clinical Medicine, Aarhus University, Aarhus, Denmark; 2 Department of Obstetrics and Gynecology, Copenhagen University Hospital, Hvidovre, Denmark

**Keywords:** Pregnancy, Mental disorders, Mental health, Psychosocial vulnerability, Mindfulness-Based Stress Reduction, MBSR, Mindfulness, Self-compassion, Motivational interviewing

## Abstract

**Background:**

Stress and mental disorders in pregnancy can adversely affect the developing fetus. Women with a preconception history of mental disorders or of psychosocial vulnerabilities are at increased risk of experiencing perinatal stress or mental health problems. Mindfulness-Based-Stress-Reduction (MBSR) is an acceptable intervention for pregnant women and has a growing evidence-base with meta-analyses consistently pointing to reductions in symptoms of stress, anxiety and depression. This study protocol aim to address the need for a wider array of evidence-based and non-pharmacological options during pregnancy to reduce stress and improve mental health in a psychosocially highly vulnerable group of women.

**Methods:**

Pregnant women with a preconception history of mental disorders or psychosocial vulnerabilities (*n* = 240) will be recruited from an obstetric ambulatory clinic at Copenhagen University Hospital, Hvidovre, Denmark. Recruitment for the study began in March 2022 and will continue until the desired number of participants is reached. Consenting pregnant women will be randomized to one of two study arms, an adapted MBSR program as add on to usual care or usual care alone. The primary outcome is mental wellbeing at nine months post-randomization. Secondary and exploratory outcomes include stress, anxiety, depression, and maternal antenatal attachment, experience of childbirth, delivery and mode of delivery. Mindfulness and self-compassion are examined as possible mediators of the effect on outcomes.

**Discussion:**

Teaching the skills of mindfulness meditation to a psychosocially vulnerable group of pregnant women could prove a viable and non-pharmacological approach to improve mental health and wellbeing during pregnancy, reduce stress and support the transition to parenthood. Mindfulness-Based Stress Reduction does not target a particular group, and results from the study is thus of potential relevance for pregnant women in general as a means of reducing stress and improving perinatal mental health and wellbeing.

**Trial registration:**

ClinicalTrials.gov: NCT05300646. Registered March 29, 2022.

## Background

Entering motherhood is one of the most intensive transitional stages in life [1, 2]. For women with a preconception history of mental disorders and psychosocial vulnerability, pregnancy can be a time of increased susceptibility to stress, anxiety or depression when coping with the new event of becoming a mother [2, 3]. The term psychosocial refers to psychological and social factors that influence mental health [4], and is used here as a collective term for vulnerabilities that may pose a risk to maternal mental health or the health of the unborn child. This includes, but is not limited to, a history of childhood abuse, low social support, and intimate partner violence, past trauma, current affective disorders or poor adaptation to pregnancy. Perinatal maternal stress and mental disorders are adversely associated with early life development of the child [5–8], and increased all-cause mortality risk in adulthood [9]. Pregnancy is thus an important window of opportunity for preventive and therapeutic interventions. However, there is a need for more research on the effectiveness of non-pharmacological interventions [2, 10], and in particular for highly vulnerable groups, which has informed the design of this study.

Mindfulness-Based Stress Reduction (MBSR) is a group-based therapy aimed at strengthening mental health and supporting resilience through the cultivation of mindfulness and compassion [11]. MBSR is not a conventional group therapy, but offers a practical educational approach involving the creation of a highly participatory community of participants intent on learning and integrating into their lives a different way of being [12]. Mindfulness-Based Interventions (MBIs) including MBSR are acceptable for pregnant women with a history of mental disorders [10], and offers approaches for addressing transdiagnostic factors, e.g. meta-awareness, present-centered awareness and nonreactivity [13]. In sum, MBSR may be suitable for a clinical context that provides care for highly vulnerable pregnant women. Moreover, MBSR has a growing evidence-base with meta-analyses and systematic reviews consistently pointing to significant reductions in symptoms of stress, anxiety and depression, and to improvements in mental wellbeing in both clinical and non-clinical populations [14–16]. Compared to first-line, evidence-based therapies such as cognitive behavioral therapy or psychotropic medication, MBIs are equally efficacious for depression [17, 18]. While promising results have been reported for anxiety when compared to evidence-based, therapies more research is needed [15, 17, 19]. Given the growing evidence, interest in the mechanisms of action are increasing [20–22]. Consistent evidence has been identified for mindfulness [20–23] and to some degree for self-compassion [20, 22, 23] as possible mediators of the effect of training the mind. Systematic reviews and meta-analyses on the effectiveness of MBIs on depression, anxiety and stress during pregnancy in both clinical and non-clinical populations have also increased rapidly in recent years [24–28]. One systematic review and meta-analysis examined the effect of MBIs on clinical and subthreshold perinatal depression and anxiety [24]. A strength of the meta-analysis was a thorough assessment of symptom levels at baseline. Results showed a significant and lasting effect on symptoms of perinatal depression in both groups when compared to both active and non-active controls. The effect on anxiety was also significant, but considered preliminary due to the current unavailability of high quality research. Importantly, no significant difference on effect was found when MBIs were delivered in person or through digital means [24], which could help remove barriers to treatment. Another systematic review and meta-analysis focusing on perinatal depression alone showed that MBIs delivered during pregnancy significantly mitigated the severity of depressive symptoms up to three months after childbirth, but that the effect tended to diminish after six months [25]. The authors argued that continuing the intervention into the early postpartum period may be necessary to help sustain the effect. A meta-analysis examined the effect of MBIs delivered during pregnancy on mental health and in particular on postpartum depression three months after childbirth [28]. This meta-analysis differed from the above in that both Chinese and English literature were included. Significant effects on postpartum depressive symptoms in pregnant women with no history of mental disorders were shown, whereas no clear effects were shown for women with a history of mental disorders or symptoms of anxiety and depression during pregnancy [28]. A systematic review and meta-analysis evaluated the effect of psychological interventions including MBIs on perinatal anxiety, and while MBIs demonstrated promising effects on perinatal anxiety and comorbid anxiety and depression, the evidence is preliminary [26]. In sum, the evidence for an effect of MBI´s on perinatal depression is increasingly robust and in line with the evidence in general. While promising, evidence for an effect on perinatal anxiety and stress is less robust and call for more research.

Parenting begins in pregnancy. Maternal antenatal attachment is a term used to describe the emotional bond between a pregnant woman and her unborn child [29]. Mindfulness may improve relational functioning [30] and could thus possibly strengthen the maternal bonding process during pregnancy. So far, research including maternal antenatal attachment as an outcome is limited. One RCT reported a significant improvement in maternal fetal attachment in pregnant women after participation in a Mindfulness-Based Childbirth and Parenting program (MBCP), which is based on MBSR [31]. The study did not include women with a history of mental disorders, however. While no firm conclusions can be drawn about the future quality of parenting based on maternal attachment during pregnancy [32], it is nonetheless a relevant outcome to explore further in this context.

Fear of childbirth is more prevalent in women with few psychological and social resources when compared to a general population [33], and has been associated with emergency caesarian section and prolonged labor [34]. A small randomized controlled trial (RCT) aimed at reducing fear of childbirth in pregnant women examined the effect of a short version of MBCP [35]. When compared to standard childbirth education, the study suggested that mindfulness training is a promising avenue for preparing women for childbirth [35]. Another, small study also reported positive findings on fear of childbirth after participation in a MBI [36], and one RCT reported lower rates of caesarian section deliveries among pregnant women after participating in an adapted MBCT program, and an indirect effect on gestational age [37]. Moreover, patient reported satisfaction is important when aiming to implement new interventions in hospital-based settings [38], and childbirth experience is thus a relevant measure to include.

Engaging in MBSR is time consuming and requires both great motivation and commitment from the participant. Motivation for change is often high during pregnancy, but the availability of resources may nevertheless be lower in psychosocially vulnerable groups and increase the risk for premature dropout from the intervention. Motivational interviewing (MI) is an evidence-based psychotherapeutic approach that focuses on engaging, encouraging and facilitating intrinsic motivation within the individual to promote behavior change [39]. One study that applied MI in connection with a MBI with the purpose of increasing adherence and reducing dropout reported a significant impact on intervention adherence and dropout rates when compared to a group not undergoing MI [40]. MI will therefore be included in the present study with the aim of increasing adherence and reducing dropout.

### Study aims

The overall aim of the study is to address the need for a wider array of evidence-based and non-pharmacological options to reduce stress and support resilience in pregnant women with a preconception history of mental disorders or psychosocial vulnerabilities. The intention of mindfulness meditation training in this context is twofold: (1) to improve mental wellbeing and reduce symptoms of stress, anxiety and depression, (2) to engage and strengthen pregnant women´s internal resources for coping more effectively with the challenges and demands of pregnancy and motherhood including building a positive relation to the child. In more detail, the primary aim of the RCT is to estimate the effect of prenatal MBSR as an add-on to usual care on mental wellbeing in psychosocially vulnerable pregnant women when compared to usual care alone. Second, to estimate the effect of prenatal MBSR on perceived stress, symptoms of depression and anxiety. Third to examine if mindfulness and self-compassion mediate an effect of the intervention on outcomes and fourth, to explore the effect on maternal antenatal attachment and childbirth, e.g. gestational age at birth and experience of the birth. To fulfill these aims the MBSR program was adapted to fit a clinical context and named prenatal MBSR [41].

### Design

This protocol adheres to the guidance of the Standard Protocol Items: Recommendations for Interventional Trials (SPIRIT) statement for reporting randomized clinical trials [42]. The design is a single-center, parallel group, randomized controlled trial comparing prenatal MBSR as add on to usual care (UC) with usual care alone. Participants are randomized in a 1:1 ratio to an intervention group or a waitlist control group receiving care as usual. Participants in the waitlist control group are offered participation in an equivalent MBSR program for mother and baby after the follow-up period. An outline of the study timeline is shown in Table 1.

**Table 1:** Participant timeline

Timeline	Event
First consultation	Interest in study participation and preliminary eligibility is established at first consultation at the obstetric outpatient clinic (around 18 weeks gestation), and written information is emailed to possible participants.
− 2 weeks	Interview to determine eligibility and participation within two weeks from first consultation.
− 3 weeks	Motivational interviewing (MI)
	Baseline questionnaire
0	Randomization
+ 2 weeks	Start of the intervention (9 weeks)
T1 (1½ months)	1½ months post-randomization: second and intermediate assessment
T2 (3 months)	3 months post-randomization: Third assessment
T3 (6 months)	6 months post-randomization: Fourth assessment
T4 (9 months)	9 months post-randomization: Fifth and final assessment

## Methods

### Setting

The study is carried out at an obstetric ambulatory clinic at Copenhagen University Hospital, Hvidovre, which is the largest birthplace in Denmark with 7000 annual births. The obstetric ambulatory clinic has on average 750 referrals per year and is one of five ambulatory clinics in Denmark, which provide antenatal care for highly vulnerable pregnant women. Reasons for referral to the obstetric ambulatory clinic is a preconception history of one or more mental disorders irrespective of illness stage at the time of referral and/or complicating psychosocial vulnerabilities. Psychosocial vulnerabilities include, but not limited to, a history of childhood adversity, sexual assaults, present physical or psychological intimate partner violence, past or current substance abuse, current affective disorders or poor adaptation to pregnancy. In the majority of cases (> 60%) the reason for referral include a preconception history of one or more mental disorders. The purpose of the antenatal care as determined by the Danish Health Authority is to reduce the risk for obstetric complications and to promote a healthy pregnancy, including maternal antenatal attachment. Due to the complexity, the obstetric ambulatory clinic works multidisciplinary and include midwifes, physicians, social workers and psychologists. In Denmark, there is free and equal access to most healthcare services including hospitals, general practitioners and practicing specialists. General practitioners act as the gateway to all healthcare services.

### Recruitment and consent

Recruitment for the study takes place at the first consultation with a midwife at the obstetric ambulatory clinic, which is on average at 18 week’s gestation. Preliminary eligibility for the study is determined in a collaboration between the midwife and the pregnant woman based on her medical record, reason for referral to the ambulatory clinic and her present surplus of mental resources. The midwife will provide verbal information about the study, and email written information to eligible women, who express an interest in participating in the study. Name, contact information and reason for referral is then passed on to the responsible researcher, who will schedule an interview. The interview will take place either online or at the outpatient clinic. The online format provides the women with an opportunity to participate in the interview in their own homes. Our experience working with this population, led to offering this option. We offer the possibility of conducting the interview at the outpatient clinic if it is not possible online. The interview contains information about the study, eligibility criteria, procedures, randomization and the intervention. Inclusion criteria for the study are estimated due date no sooner than three months from start of the intervention, eighteen + years of age, able to speak and write Danish and written informed consent to study criteria. Exclusion criteria are an active substance dependence, psychotic disorders (e.g. schizophrenia or bipolar disorder), suicidality and being unavailable for two or more of the scheduled prenatal MBSR classes. Pregnant women who fulfill the eligibility criteria and consent to study participation must sign a consent form no later than one week from the interview. The responsible researcher (first author) obtains written informed consent.

### Study – and intervention adherence

Engaging in a MBSR program is time consuming and require both motivation and commitment from the participant. Motivation is an important factor in treatment adherence and retention in general and high dropout rates is a well-documented problem in particular group therapies [40]. Motivational interviewing (MI) is an evidence-based psychotherapeutic approach that focuses on engaging, encouraging and facilitating intrinsic motivation within the individual to promote behavior change [39]. Motivation for change is often high during pregnancy, but drop- out rates may nevertheless be high because of the increased demand for both physical and mental resources. Premature dropout may result in participants not receiving the full benefit of the intervention. In order to prepare the pregnant women for engaging in prenatal MBSR and reduce dropout, MI will be applied in the present study. The interviews, which will take place prior to randomization, will be conducted individually and in a semi-structured manner by the responsible researcher. Each interview is scheduled to last 30 min.

### Intervention: the prenatal MBSR program

The prenatal MBSR program is an adaptation of MBSR in order to fit into a clinical context. Importantly, the essential “warp” elements characterizing MBSR were maintained [43]. The adaptation process is described in detail in Skovbjerg S et al. [41]. In brief, the prenatal MBSR program include nine weekly two-hour classes with a maximum of 15 participants per group. The program is delivered in a combination between physical attendance (class 1, 4 and 9) and live-online teaching. The intention of combining physical attendance with live-online teaching is to reduce travelling and ensure attendance while still providing a good starting point for establishing a learning environment and a sense of community in the group. Pregnancy and motherhood are a natural focus throughout the program, e.g. sitting meditations include periods sitting with one hand on the pregnant belly and the other hand on the heart. Yoga programs suitable for pregnancy was developed. The recommended time for daily mindfulness training between classes is 15 min a day with options for longer practice. This in order to make the course requirements manageable considering the psychosocial vulnerabilities of the women. Participants receive an email after each class summarizing key points from the training and instructions for home practice. Audio recordings with guided meditations and videos with the yoga programs for pregnancy are provided for home practice. Participants are asked to register the time they spent on formal mindfulness practice between classes. The data will be collected after the final class. Participation is registered for each class and participants are encouraged to notify the teacher in case of non-attendance. Participants will be contacted by phone in cases of absence from two successive classes or in cases where the teacher is not notified about non-attendance.

A certified MBSR teacher with a professional background in psychiatry (last author, Lone Fjorback) teach the prenatal MBSR program. Three midwifes employed at the obstetric ambulatory clinic, who are training to become MBSR teachers, co-teach the prenatal MBSR program. Only one midwife at a time is a co-teacher for a group.

An online booster session is offered three months after finalizing the 9-week program where the new mothers are invited to bring their babies and thus making it an opportunity to practice being mindful with their baby.

### Control condition: usual care

Standard clinical practice, usual care, include an average of six routine pregnancy consultations with a midwife at the obstetric ambulatory clinic. If needed, additional visits is scheduled that may include consultations with a physician, a social worker or a psychologist.

### Outcomes

Primary, secondary and exploratory outcomes include validated self-report questionnaires and data from medical records. Questionnaires are collected at the following five time points in both the intervention and waitlist control group: at baseline, prior to randomization (T0), 1½ months post-randomization (T1), 3 months post-randomization (T2), 6 months post-randomization (T3) and 9 months post-randomization (T4). The questionnaire packet is built and managed via REDCap (Research Electronic Data Capture) hosted by Aarhus University. REDCap is a secure web application for building and managing online surveys. In case of non-response, reminders are emailed after one week. Table 2 presents an overview of included questionnaires and time points for assessments.

**Table 2:** Overview of self-report questionnaires and time points for assessments

Questionnaire/assessment	Latent variable	T0	T1	T2	T3	T4
***Primary outcome measure***						
The Who – Well-Being Index	Mental well being	X	X	X	X	X
***Secondary outcome measures***						
The Edinburgh Depression Scale	Depression	X		X	X	X
Depression, Anxiety, Stress scales	Distress	X		X	X	X
***Exploratory outcome measures***						
Maternal Antenatal Attachment Scale	Antenatal attachment	X		X		
The Childbirth Experience Questionnaire	Childbirth				X	
***Mediators of outcome***						
The Five-Facet Mindfulness Questionnaire	Mindfulness	X	X	X	X	X
The Self-Compassion Scale	Self-compassion	X	X	X	X	X

T0 = Baseline; T1 = 1½ months post-randomization; T2 = 3 months post-randomization; T3 = 6 months post-randomization; T4 = 9 months post-randomization

### Primary outcome measure

#### The World Health Organization – Five Well-Being Index

The World Health Organization – Five Well-Being Index (WHO-5) is a short and generic global rating scale measuring subjective wellbeing [44]. It consists of five statements and the respondent is asked to rate how well each of the statements applies to her when considering the last 14 days. Each item is scored from 5 “all of the time” to 0 “none of the time”. Final scores range from 0 to 100 with higher scores representing greater wellbeing. The scale has adequate validity as an outcome measure in clinical trials and has been applied successfully across a wide range of study fields [44].

### Secondary outcome measures

#### Depression Anxiety Stress Scales

The Depression Anxiety Stress Scales (DASS-21) has three subscales designed to discriminate between depression, anxiety and stress in the last week [45]. The DASS excludes somatic items such as sleep disturbance, lack of energy and poor concentration, which may not be valid markers in pregnancy or the postpartum period [46]. Each subscale includes seven items. Response to each item is rated on a four-point Likert scale ranging from ‘never’ to ‘very much/most of the time’. Scores are calculated for each subscale and higher scores point to more symptoms of depression, anxiety or stress. The DASS-21 has shown good psychometric properties [47].

#### The Edinburgh Depression Scale

The Edinburgh Depression Scale (EDS) is a widely used screening questionnaire for depression in the perinatal period. The EDS contains 10 questions on how the respondent has felt in the past seven days with each item scored 0–3 yielding a maximum score of 30 [48]. A higher score points to more depressive symptoms, and a score of 11 has been suggested as the optimal cutoff for depression according to both DSM-5 and ICD-10 criteria [49].

### Exploratory outcome measures

#### The Maternal Antenatal Attachment Scale

The Maternal Antenatal Attachment Scale (MAAS) is a widely used measure of the maternal-fetal bond [50]. The maternal-fetal bond has been defined as “the emotional tie or bond which normally develops between the pregnant woman and her unborn child” [51]. The theoretical basis of MAAS is a hierarchical model of adult attachment [50]. Within this model, the core experience of maternal attachment is the mothers love for her unborn child. The MAAS consists of 19 items covering two subscales: 1) Quality of attachment (11 items) and time spent in attachment mode (8 items) [52]. All items are rated on a five-point scale. The minimum score for the total MAAS is 19 and the maximum is 95. The scores for the subscales range between 11 and 50 (Quality of attachment) and between 8 and 40 (Time spent in attachment mode). Higher scores reflect, respectively, a positive quality of attachment and a high intensity of preoccupation with the fetus [52]. Moreover, MAAS scores can be divided into four maternal attachment styles based on mean scores on the two subscales [53]. The MAAS has demonstrated good psychometric properties in terms of structural validity and internal consistency [54].

#### The Childbirth Experience Questionnaire

The Childbirth Experience Questionnaire (CEQ) assess different aspects of childbirth experience [55]. The CEQ contains 22 statements assessing four domains of the childbirth experience; own capacity, professional support, participation, and perceived safety. For 19 of the items the response format is a 4-point Likert Scale whereas the last three items use a visual analogue scale (VAS). Higher scores reflect a more positive birth experience. The CEQ has demonstrated an acceptable construct validity and reliability in a Danish setting [38].

### Mediator variables

#### The Five-Facet Mindfulness Questionnaire

The Five-Facet Mindfulness Questionnaire (FFMQ) assesses five general facets of being mindful in daily life: observing, describing, acting with awareness, non-reactivity to inner experience, and non-judging of inner experience. Items are rated on a five-point Likert scale ranging from 1 “never or very rarely true” to 5 “very often or always true”. Higher scores suggest higher levels of mindfulness. Previous studies have provided good support for the construct validity of the FFMQ [56].

#### The Self-Compassion Scale

The Self-Compassion Scale (SCS) measures the ability to have a healthy stand towards oneself that does not involve evaluations of self-worth [57]. The scale consists of 12 items and responses are given on a 5-point scale from ranging from 1 “almost never” to 5 “almost always”. Higher scores indicate more self-compassionate behavior. The psychometric properties of the scale has been extensively evaluated [57].

#### Participant characteristics

Baseline data on estimated due date, age, marital status, number of children, social support, working status, education, obstetric complications (e.g. gestational diabetes), current use of psychotropic medication, therapy and experience with mindfulness-based interventions will be collected by means of a self-report questionnaire. Data on history of mental disorders and psychosocial vulnerabilities, and data on time and mode of delivery, birth length and weight will be collected from the participants’ medical record.

### Sample size

The primary outcome is change in mental well-being (WHO-5) from baseline to post-intervention. The aim is to include 240 psychosocially vulnerable pregnant women in the period from March 2022 and until the desired number has been reached. About 750 women are referred to the obstetric ambulatory clinic every year and the time span is therefore considered realistic. Out of 240 pregnant women, 168 (70%) is expected to complete the study based on the dropout rate in the feasibility trial [41]. With this sample size, a standard deviation of 15.6 (data from the feasibility trial) and a two-sided alpha of 5%, the study has 82% power to detect a difference of 7 on the WHO-5 between the two study groups, and 91% power to detect a difference of 8 on the WHO-5.

### Randomization

The study will use the REDCap randomization module hosted by Aarhus University for the randomization procedure. To secure concealment, a data manager from Aarhus University, not otherwise involved in the study, programed the randomization algorithm. Enrollment and randomization is conducted by the responsible researcher, and takes place after the statement of consent has been signed and the first questionnaire has been filled in. Participants are randomized in a 1:1 ratio. Inclusion and thus randomization will take place consecutively.

### Criteria for discontinuing allocated interventions

Criteria for discontinuation of study participation is if requested by the participant.

### Statistical analysis plan

Statistical analyses will be performed using SPSS for Windows (version 28; IBM Corp, Armonk, NY) or Stata (version 16). Recruitment, participation and retention rates will be reported in a CONSORT flow diagram [58]. Analyses on the primary outcome will be conducted on the intent-to-treat population meaning all randomized participants with completed baseline assessments. Compliance is considered as participation in five or more of intervention-scheduled classes.

Baseline sociodemographic data will be presented by means of frequencies (percentages), medians (interquartile range), and means (SD), according to their level of measurement and statistical distribution.

Statistical analysis of primary outcome, mental wellbeing, will be performed using a repeated measurement model with systematic effects, randomization arm, time (baseline, 3, 6 and 9 months post-randomization), intervention (prenatal MBSR, waitlist control group), interaction between time and intervention, baseline values of: age, perceived stress and use of psychotropic drugs. The model will include random effects of person and “scheduled group”. The primary effect estimate is the mean difference in change since baseline between the intervention group and the control group at nine months post-randomization. The change from baseline at 3 and 6 months will also be reported. Equivalent models will analyze secondary outcomes, stress, anxiety and depression. The change from baseline to three months after randomization in maternal antenatal attachment will be analyzed in a model with the systematic effect of intervention, baseline mental wellbeing, perceived stress, age, number of children and random effect of “scheduled group”. Equivalent models will be used in the analysis of experience of childbirth. A per-protocol analysis of the primary outcome will be performed with three intervention levels: (1) attending five or more of intervention-scheduled sessions, (2) attending less than five of intervention scheduled sessions and (3) waitlist control group. No subgroup analyses is planned. Standard errors will be based on the bootstrap method, resampling persons.

The five measurement points allow to test whether changes in the proposed mediators, i.e. mindfulness and self-compassion, are associated with changes in the proposed outcomes. Structural equation modelling will be used for the statistical analyses of the proposed mechanisms [59].

Four sensitivity analyses is planned to evaluate the size of bias if data not being missing at random. In each of the four analyses, missing outcomes after baseline will be imputed as the model based estimated value when using the observed data, plus a systematic deviation. First, the deviation will be zero in the waitlist control group and + 0.2*SD in the prenatal MSBR group. Second, the deviation will be zero in the waitlist control group and − 0.2*SD in the prenatal MSBR group, and then + 0.2*SD in the waitlist control group and zero in the prenatal MSBR group in the third analysis. In the fourth and final analysis, the deviation will be -0.2*SD in the waitlist control group and zero in the prenatal MSBR group.

### Blinding

Group allocation will not be blinded since keeping participants blind to this type of intervention would not be possible.

### Scientific ethical considerations

Participation in a mindfulness-based program has not been associated with an increased incidence of harm when compared to no treatment [60]. The most common experiences of harm or adverse effects of meditation including mindfulness-meditation is anxiety, traumatic re-experiencing and emotional sensitivity [61]. However, childhood adversity has been associated with a higher frequency of adverse effects [61], which emphasize the need for trauma sensitivity in the present context. A psychiatrist with extensive clinical psychiatric experience will be teaching the prenatal MBSR program. If any adverse events should occur, the teacher has the relevant expertise to ensure participant safety or referral to relevant treatment elsewhere. The teacher and co-teacher are available for individual sessions between the program sessions if needed. Any side effects related to the intervention will be registered and published with the results of the study. The possible benefits of mindfulness training in pregnancy as described in earlier sections thus outweighs the potential side effects of the intervention. Moreover, there is a need for more evidence-based, non-pharmacological options for addressing prenatal mental healthcare needs in vulnerable groups for the benefit of both mother and baby. The adaptations made to the original MBSR program require more research in order to establish an evidence-base for the prenatal MBSR program, which is the aim of this study.

## Discussion

The intention of mindfulness meditation training for vulnerable pregnant women is to improve mental wellbeing, reduce symptoms of stress, anxiety and depression, and to engage and strengthen the women´s internal resources for coping more effectively with the challenges and demands of pregnancy and motherhood. The skills inherent in the MBSR program, e.g. present moment awareness, self-regulation and meta-cognition, are vital in improving mental health and coping with the stress that may be part of the transition to parenthood. Moreover, they can support the formation of a healthy attachment to the unborn child. MBSR does not target a particular group but can be applied to pregnant women in general and when there is a need to support mental wellbeing and provide skills for effective stress management. The results from the present study is thus of potential relevance to maternity care in general.

## Data Availability

Not applicable as no dataset have been generated.

## Abbreviations

MBSR: Mindfulness-Based Stress Reduction
MBI: Mindfulness-based interventions
MBCP: Mindfulness-Based Childbirth and Parenting program
RCT: Randomized Controlled Trial
WHO-5: The World Health Organization - Five Well-Being Index
DASS-21: Depression Anxiety Stress Scales - 21
EDS: The Edinburgh Depression Scale
MAAS: The Maternal Antenatal Attachment Scale
CEQ: The Childbirth Experience Questionnaire
FFMQ: The Five-Facet Mindfulness Questionnaire
SCS: Self-Compassion Scale
